# Skellam Type Processes of Order *k* and Beyond

**DOI:** 10.3390/e22111193

**Published:** 2020-10-22

**Authors:** Neha Gupta, Arun Kumar, Nikolai Leonenko

**Affiliations:** 1 Department of Mathematics, Indian Institute of Technology Ropar, Rupnagar, Punjab 140001, India; 2017maz0002@iitrpr.ac.in (N.G.); arun.kumar@iitrpr.ac.in (A.K.); 2 Cardiff School of Mathematics, Cardiff University, Senghennydd Road, Cardiff CF24 4AG, UK

**Keywords:** Skellam process, subordination, Lévy measure, Poisson process of order k, running average

## Abstract

In this article, we introduce the Skellam process of order *k* and its running average. We also discuss the time-changed Skellam process of order *k*. In particular, we discuss the space-fractional Skellam process and tempered space-fractional Skellam process via time changes in Skellam process by independent stable subordinator and tempered stable subordinator, respectively. We derive the marginal probabilities, Lévy measures, governing difference-differential equations of the introduced processes. Our results generalize the Skellam process and running average of Poisson process in several directions.

## 1. Introduction

The Skellam distribution is obtained by taking the difference between two independent Poisson distributed random variables, which was introduced for the case of different intensities $\lambda_{1},\;\lambda_{2}$ by (see [1]) and for equal means in [2]. For large values of $\lambda_{1}+\lambda_{2}$, the distribution can be approximated by the normal distribution and if $\lambda_{2}$ is very close to 0, then the distribution tends to a Poisson distribution with intensity $\lambda_{1}$. Similarly, if $\lambda_{1}$ tends to 0, the distribution tends to a Poisson distribution with non-positive integer values. The Skellam random variable is infinitely divisible, since it is the difference of two infinitely divisible random variables (see Proposition 2.1 in [3]). Therefore, one can define a continuous time Lévy process for Skellam distribution, which is called Skellam process.

The Skellam process is an integer valued Lévy process and it can also be obtained by taking the difference of two independent Poisson processes. Its marginal probability mass function (pmf) involves the modified Bessel function of the first kind. The Skellam process has various applications in different areas, such as to model the intensity difference of pixels in cameras (see [4]) and for modeling the difference of the number of goals of two competing teams in a football game [5]. The model based on the difference of two point processes is proposed in (see [6,7,8,9]).

Recently, the time-fractional Skellam process has been studied in [10], which is obtained by time-changing the Skellam process with an inverse stable subordinator. Further, they provided the application of time-fractional Skellam process in modeling of arrivals of jumps in high frequency trading data. It is shown that the inter arrival times between the positive and negative jumps follow Mittag–Leffler distribution rather then the exponential distribution. Similar observations are observed in the case of Danish fire insurance data (see [11]). Buchak and Sakhno, in [12], have also proposed the governing equations for time-fractional Skellam processes. Recently, [13] introduced time-changed Poisson process of order *k*, which is obtained by time changing the Poisson process of order *k* (see [14]) by general subordinators.

In this paper, we introduce Skellam process of order *k* and its running average. We also discuss the time-changed Skellam process of order *k*. In particular, we discuss space-fractional Skellam process and tempered space-fractional Skellam process via time changes in Skellam process by independent stable subordinator and tempered stable subordinator, respectively. We obtain closed form expressions for the marginal distributions of the considered processes and other important properties. Skellam process is used to model the difference between the number of goals between two teams in a football match. At the beginning, both teams have scores 0 each and at time *t* the team 1 score is $N_{1}\left(t\right)$, which is the cumulative sum of arrivals (goals) of size 1 until time *t* with exponential inter-arrival times. Similarly for team 2, the score is $N_{2}\left(t\right)$ at time t. The difference between the number of goals can be modeled using $N_{1}\left(t\right)-N_{2}\left(t\right)$ at time *t*. Similarly, the Skellam process of order *k* can be used to model the difference between the number of points scored by two competing teams in a basketball match where k=3. Note that, in a basketball game, a free throw is count as one point, any basket from a shot taken from inside the three-point line counts for two points and any basket from a shot taken from outside the three-point line is considered as three points. Thus, a jump in the score of any team may be of size one, two, or three. Hence, a Skellam process of order 3 can be used to model the difference between the points scored.

In [10], it is shown that the fractional Skellam process is a better model then the Skellam process for modeling the arrivals of the up and down jumps for the tick-by-tick financial data. Equivalently, it is shown that the Mittag–Leffler distribution is a better model than the exponential distribution for the inter-arrival times between the up and down jumps. However, it is evident from Figure 3 of [10] that the fractional Skellam process is also not perfectly fitting the arrivals of positive and negative jumps. We hope that a more flexible class of processes like time-changed Skellam process of order *k* (see Section 6) and the introduced tempered space-fractional Skellam process (see Section 7) would be better model for arrivals of jumps. Additionally, see [8] for applications of integer-valued Lévy processes in financial econometrics. Moreover, distributions of order *k* are interesting for reliability theory [15]. The Fisher dispersion index is a widely used measure for quantifying the departure of any univariate count distribution from the equi-dispersed Poisson model [16,17,18]. The introduced processes in this article can be useful in modeling of over-dispersed and under-dispersed data. Further, in (49), we present probabilistic solutions of some fractional equations.

The remainder of this paper proceeds, as follows: in Section 2, we introduce all the relevant definitions and results. We also derive the Lévy density for space- and tempered space-fractional Poisson processes. In Section 3, we introduce and study running average of Poisson process of order *k*. Section 4 is dedicated to Skellam process of order *k*. Section 5 deals with running average of Skellam process of order *k*. In Section 6, we discuss the time-changed Skellam process of order *k*. In Section 7, we determine the marginal pmf, governing equations for marginal pmf, Lévy densities, and moment generating functions for space-fractional Skellam process and tempered space-fractional Skellam process.

## 2. Preliminaries

In this section, we collect relevant definitions and some results on Skellam process, subordinators, space-fractional Poisson process, and tempered space-fractional Poisson process. These results will be used to define the space-fractional Skellam processes and tempered space-fractional Skellam processes.

### 2.1. Skellam Process

In this section, we revisit the Skellam process and also provide a characterization of it. Let S(t) be a Skellam process, such that

$$S\left(t\right)=N_{1}\left(t\right)-N_{2}\left(t\right),\;t\geq 0,$$

where $N_{1}\left(t\right)$ and $N_{2}\left(t\right)$ are two independent homogeneous Poisson processes with intensity $\lambda_{1}>0$ and $\lambda_{2}>0,$ respectively. The Skellam process is defined in [8] and the distribution has been introduced and studied in [1], see also [2]. This process is only symmetric when $\lambda_{1}=\lambda_{2}$. The pmf $s_{k}\left(t\right)=P\left(S\left(t\right)=k\right)$ of S(t) is given by (see e.g., [1,10])

(1) $$\begin{array}{c} s_{k}\left(t\right)=e^{-t(\lambda_{1}+\lambda_{2})}{\left(\frac{\lambda_{1}}{\lambda_{2}}\right)}^{k/2}I_{\left|k\right|}\left(2t\sqrt{\lambda_{1}\lambda_{2}}\right),\;k\in Z, \end{array}$$

where $I_{k}$ is modified Bessel function of first kind (see [19], p. 375),

(2) $$I_{k}\left(z\right)=\sum_{n=0}^{\infty }\frac{{\left(z/2\right)}^{2n+k}}{n!(n+k)!}.$$

The pmf $s_{k}\left(t\right)$ satisfies the following differential difference equation (see [10])

(3) $$\frac{d}{dt}s_{k}\left(t\right)=\lambda_{1}\left(s_{k-1}\left(t\right)-s_{k}\left(t\right)\right)-\lambda_{2}\left(s_{k}\left(t\right)-s_{k+1}\left(t\right)\right),\;\;k\in Z,$$

with initial conditions $s_{0}\left(0\right)=1$ and $s_{k}\left(0\right)=0,\;k\neq 0$. For a real-valued Lévy process Z(t) the characteristic function admits the form

(4) $$E\left(e^{iuZ(t)}\right)=e^{t\psi_{Z}\left(u\right)},$$

where the function $\psi_{Z}$ is called characteristic exponent and it admits the following Lévy-Khintchine representation (see [20])

(5) $$\psi_{Z}\left(u\right)=iau-bu^{2}+\int_{R\backslash \{0\}}\left(e^{iux}-1-iux1_{\left\{|x|\leq 1\right\}}\right)\pi_{Z}\left(dx\right).$$

Here, a∈R,b≥0 and $\pi_{Z}$ is a Lévy measure. If $\pi_{Z}\left(dx\right)=\nu_{Z}\left(x\right)dx$ for some function $\nu_{Z}$, then $\nu_{Z}$ is called the Lévy density of the process Z. The Skellam process is a Lévy process, its Lévy density $\nu_{S}$ is a linear combination of two Dirac delta functions, $\nu_{S}\left(y\right)=\lambda_{1}\delta_{1}\left(y\right)+\lambda_{2}\delta_{-1}\left(y\right)$ and the corresponding characteristic exponent is given by

$$\psi_{S(1)}\left(u\right)=\int_{-\infty }^{\infty }\left(1-e^{-uy}\right)\nu_{S}\left(y\right)dy.$$

The moment generating function (mgf) of Skellam process is

(6) $$\begin{array}{c} E\left[e^{\theta S(t)}\right]=e^{-t(\lambda_{1}+\lambda_{2}-\lambda_{1}e^{\theta }-\lambda_{2}e^{-\theta })},\;\theta \in R. \end{array}$$

With the help of mgf, one can easily find the moments of Skellam process. In the next result, we give a characterization of Skellam process, which is not available in literature as per our knowledge. For a function *h*, we write h(δ)=o(δ) if $\lim_{\delta \to 0}h\left(\delta \right)/\delta =0.$

**Theorem** **1.** *Suppose that an arrival process has the independent and stationary increments and it also satisfies the following incremental condition, then the process is Skellam.*

$$P\left(S\left(t+\delta \right)=m|S\left(t\right)=n\right)=\left\{\begin{array}{cc} \lambda_{1}\delta +o\left(\delta \right), & m>n,\;m=n+1;\\ \lambda_{2}\delta +o\left(\delta \right), & m<n,\;m=n-1;\\ 1-\lambda_{1}\delta -\lambda_{2}\delta +o\left(\delta \right), & m=n;\\ o\left(\delta \right) & \mathrm{otherwise}. \end{array}\right.$$

**Proof.**  Consider the interval [0,t], which is discretized with *n* sub-intervals of size δ each, such that nδ=t. For k≥0, we have

$$\begin{array}{cc} P(S(t)=k) & =\sum_{m=0}^{\left[\frac{n-k}{2}\right]}\frac{n!}{m!(m+k)!(n-2m-k)!}{\left(\lambda_{1}\delta \right)}^{m+k}{\left(\lambda_{2}\delta \right)}^{m}{\left(1-\lambda_{1}\delta -\lambda_{2}\delta \right)}^{n-2m-k}+o\left(\delta \right)\\ \; & =\sum_{m=0}^{\left[\frac{n-k}{2}\right]}\frac{n!}{m!(m+k)!(n-2m-k)!}{\left(\frac{\lambda_{1}t}{n}\right)}^{m+k}{\left(\frac{\lambda_{2}t}{n}\right)}^{m}{\left(1-\frac{\lambda_{1}t}{n}-\frac{\lambda_{2}t}{n}\right)}^{n-2m-k}+o\left(\delta \right)\\ \; & =\sum_{m=0}^{\left[\frac{n-k}{2}\right]}\frac{{\left(\lambda_{1}t\right)}^{m+k}{\left(\lambda_{2}t\right)}^{m}}{m!(m+k)!}\frac{n!}{\left(n-2m-k\right)!n^{2m+k}}{\left(1-\frac{\lambda_{1}t}{n}-\frac{\lambda_{2}t}{n}\right)}^{n-2m-k}+o\left(\delta \right)\\ \; & =e^{-(\lambda_{1}+\lambda_{2})t}\sum_{m=0}^{\infty }\frac{{\left(\lambda_{1}t\right)}^{m+k}{\left(\lambda_{2}t\right)}^{m}}{m!(m+k)!}, \end{array}$$

by taking n→∞. The result follows now by using the definition of modified Bessel function of first kind $I_{k}$. Similarly, we prove when k<0. □

### 2.2. Poisson Process of Order *k* (PPoK)

In this section, we recall the definition and some important properties of Poisson process of order k (PPoK). Kostadinova and Minkova (see [14]) introduced and studied the PPoK. Let $x_{1},x_{2},\cdots ,x_{k}$ be non-negative integers and $\zeta_{k}=x_{1}+x_{2}+\cdots +x_{k},\;\Pi_{k}!=x_{1}!x_{2}!\cdots x_{k}!$ and

(7) $$\Omega \left(k,n\right)=\{X=\left(x_{1},\;x_{2},\;\cdots ,\;x_{k}\right)|x_{1}+2x_{2}+\cdots +kx_{k}=n\}.$$

Additionally, let ${\left\{N^{k}\left(t\right)\right\}}_{t\geq 0},$ represent the PPoK with rate parameter λt, then probability mass function (pmf) is given by

(8) $$p_{n}^{N^{k}}\left(t\right)=P\left(N^{k}\left(t\right)=n\right)=\sum_{X=\Omega (k,n)}e^{-k\lambda t}\frac{{\left(\lambda t\right)}^{\zeta_{k}}}{\Pi_{k}!}.$$

The pmf of $N^{k}\left(t\right)$ satisfies the following differential-difference equations (see [14])

(9) $$\begin{array}{cc} \frac{d}{dt}p_{n}^{N^{k}}\left(t\right) & =-k\lambda p_{n}^{N^{k}}\left(t\right)+\lambda \sum_{j=1}^{n\wedge k}p_{n-j}^{N^{k}}\left(t\right),\;\;n=1,2,\ldots \\ \frac{d}{dt}p_{0}^{N^{k}}\left(t\right) & =-k\lambda p_{0}^{N^{k}}\left(t\right), \end{array}$$

with initial condition $p_{0}^{N^{k}}\left(0\right)=1$ and $p_{n}^{N^{k}}\left(0\right)=0$ and n∧k=min{k,n}. The characteristic function of PPoK $N^{k}\left(t\right)$

(10) $$\phi_{N^{k}\left(t\right)}\left(u\right)=E\left[e^{iuN^{k}\left(t\right)}\right]=e^{-\lambda t(k-\sum_{j=1}^{k}e^{iuj})},$$

where $i=\sqrt{-1}$. The process PPoK is Lévy, so it is infinite divisible i.e., $\phi_{N^{k}\left(t\right)}\left(u\right)={\left(\phi_{N^{k}\left(1\right)}\left(u\right)\right)}^{t}.$ The Lévy density for PPoK is easy to derive and it is given by

$$\nu_{N^{k}}\left(x\right)=\lambda \sum_{j=1}^{k}\delta_{j}\left(x\right),$$

where $\delta_{j}$ is the Dirac delta function concentrated at *j*. The transition probability of the PPoK ${\left\{N^{k}\left(t\right)\right\}}_{t\geq 0}$ is also given by Kostadinova and Minkova [14],

(11) $$P\left(N^{k}\left(t+\delta \right)=m|N^{k}\left(t\right)=n\right)=\left\{\begin{array}{cc} 1-k\lambda \delta , & m=n;\\ \lambda \delta & m=n+i,i=1,2,\ldots ,k;\\ 0 & \mathrm{otherwise}. \end{array}\right.$$

The probability generating function (pgf) $G^{N^{k}}\left(s,t\right)$ is given by (see [14])

(12) $$G^{N^{k}}\left(s,t\right)=e^{-\lambda t(k-\sum_{j=1}^{k}s^{j})}.$$

The mean, variance and covariance function of the PPoK are given by

(13) $$\begin{array}{cc} E[N^{k}\left(t\right)] & =\frac{k(k+1)}{2}\lambda t;\\ \mathrm{Var}[N^{k}\left(t\right)] & =\frac{k(k+1)(2k+1)}{6}\lambda t;\\ \mathrm{Cov}[N^{k}\left(t\right),N^{k}\left(s\right)] & =\frac{k(k+1)(2k+1)}{6}\lambda \left(t\wedge s\right). \end{array}$$

### 2.3. Subordinators

Let $D_{f}\left(t\right)$ be real valued Lévy process with non-decreasing sample paths and its Laplace transform has the form

$$E\left[e^{-sD_{f}\left(t\right)}\right]=e^{-tf(s)},$$

where

$$f\left(s\right)=bs+\int_{0}^{\infty }\left(1-e^{xs}\right)\pi \left(dx\right),\;\;s>0,\;b\geq 0,$$

is the integral representation of Bernstein functions (see [21]). The Bernstein functions are $C^{\infty }$, non-negative and such that ${\left(-1\right)}^{m}\frac{d^{m}}{dx^{m}}f\left(x\right)\leq 0$ for m≥1 in [21]. Here, π denote the non-negative Lévy measure on the positive half line, such that

$$\int_{0}^{\infty }\left(x\wedge 1\right)\pi \left(dx\right)<\infty ,\;\;\pi \left(\left[0,\infty \right)\right)=\infty ,$$

and *b* is the drift coefficient. The right continuous inverse $E_{f}\left(t\right)=\inf\left\{u\geq 0:D_{f}\left(u\right)>t\right\}$ is the inverse and first exist time of $D_{f}\left(t\right)$, which is non-Markovian with non-stationary and non-independent increments. Next, we analyze some special cases of Lévy subordinators with drift coefficient b = 0, which is,

(14) $$f\left(s\right)=\left\{\begin{array}{cc} p\log(1+\frac{s}{\alpha }),\;p>0,\;\alpha >0, & (\mathrm{gamma subordinator});\\ {\left(s+\mu \right)}^{\alpha }-\mu^{\alpha },\;\mu >0,\;0<\alpha <1, & (\mathrm{tempered}\alpha \text{-}\mathrm{stable}\;\mathrm{subordinator});\\ \delta (\sqrt{2s+\gamma^{2}}-\gamma ),\;\gamma >0,\;\delta >0, & (\mathrm{inverse}\;\mathrm{Gaussian}\;\mathrm{subordinator});\\ s^{\alpha },\;0<\alpha <1, & (\alpha \text{-}\mathrm{stable}\;\mathrm{subordinator}). \end{array}\right.$$

It is worth noting that, among the subordinators given in (14), all of the integer order moments of α-stable subordinators are infinite and others subordinators have all finite moments.

### 2.4. The Space-Fractional Poisson Process

In this section, we discuss main properties of space-fractional Poisson process (SFPP). We also provide the Lévy density for SFPP, which is not discussed in the literature. The SFPP $N_{\alpha }\left(t\right)$ was introduced by (see [22]), as follows

(15) $$N_{\alpha }\left(t\right)=\left\{\begin{array}{cc} N(D_{\alpha }\left(t\right)),\;t\geq 0, & 0<\alpha <1,\\ N(t),\;t\geq 0, & \alpha =1, \end{array}\right.$$

where $D_{\alpha }\left(t\right)$ is an α-stable subordinator, which is independent of homogeneous Poisson process N(t).

The probability generating function (pgf) of this process is of the form

(16) $$\begin{array}{c} G^{N_{\alpha }}\left(s,t\right)=E\left[s^{N_{\alpha }\left(t\right)}\right]=e^{{-\lambda }^{\alpha }{\left(1-s\right)}^{\alpha }t},\;\left|s\right|\leq 1,\;\alpha \in \left(0,1\right). \end{array}$$

The pmf of SFPP is

(17) $$\begin{array}{cc} P^{\alpha }\left(k,t\right)=P\left\{N_{\alpha }\left(t\right)=k\right\} & =\frac{{\left(-1\right)}^{k}}{k!}\sum_{r=0}^{\infty }\frac{{\left(-\lambda^{\alpha }\right)}^{r}t^{r}}{r!}\frac{\Gamma (r\alpha +1)}{\Gamma (r\alpha -k+1)}\\ \; & =\frac{{\left(-1\right)}^{k}}{k!}{}_{1}\psi_{1}\left[\begin{array}{c} (1,\alpha );\\ (1-k,\alpha ); \end{array}\left(-\lambda^{\alpha }t\right)\right], \end{array}$$

where ${}_{h}\psi_{i}\left(z\right)$ is the Fox Wright function (see formula (1.11.14) in [23]). It was shown in [22] that the pmf of the SFPP satisfies the following fractional differential-difference equations

(18) $$\begin{array}{cc} \frac{d}{dt}P^{\alpha }\left(k,t\right) & =-\lambda^{\alpha }{\left(1-B\right)}^{\alpha }P^{\alpha }\left(k,t\right),\;\;\alpha \in \left(0,1\right],\;k=1,2,\ldots \end{array}$$

(19) $$\begin{array}{cc} \frac{d}{dt}P^{\alpha }\left(0,t\right) & =-\lambda^{\alpha }P^{\alpha }\left(0,t\right), \end{array}$$

with initial conditions

(20) $$P^{\alpha }\left(k,0\right)=\delta_{k,0},$$

where $\delta_{k,0}$ is the Kronecker delta function, given by

(21) $$\delta_{k,0}=\left\{\begin{array}{cc} 0, & k\geq 1,\\ 1, & k=0. \end{array}\right.$$

The fractional difference operator

(22) (1−B)α=∑j=0∞αj(−1)jBj

is defined in [24], where *B* is the backward shift operator. The characteristic function of SFPP is

(23) $$E\left[e^{iuN_{\alpha }\left(t\right)}\right]=e^{-\lambda^{\alpha }{\left(1-e^{iu}\right)}^{\alpha }t}.$$

**Proposition** **1.** *The Lévy density $\nu_{N_{\alpha }}\left(x\right)$ of SFPP is given by*

(24) νNα(x)=λα∑n=1∞(−1)n+1αnδn(x).

**Proof.**  We use Lévy-Khintchine formula (see [20]),

∫R\{0}(eiux−1)λα∑n=1∞(−1)n+1αnδn(x)dx=λα∑n=1∞(−1)n+1αneiun+∑n=0∞(−1)nαn−1=λα∑n=0∞(−1)n+1αneiun=−λα(1−eiu)α,

which is the characteristic exponent of SFPP from Equation (23). □

### 2.5. Tempered Space-Fractional Poisson Process

The tempered space-fractional Poisson process (TSFPP) can be obtained by subordinating homogeneous Poisson process N(t) with the independent tempered stable subordinator $D_{\alpha ,\mu }\left(t\right)$ (see [25])

(25) $$N_{\alpha ,\mu }\left(t\right)=N\left(D_{\alpha ,\mu }\left(t\right)\right),\;\alpha \in \left(0,1\right),\;\mu >0.$$

This process have finite integer order moments due to the tempered α-stable subordinator. The pmf of TSFPP is given by (see [25])

(26) Pα,μ(k,t)=(−1)ketμα∑m=0∞μm∑r=0∞(−t)rr!λαr−mαrmαr−mk=etμα(−1)kk!∑m=0∞μmλ−mm!1ψ1(1,α);(1−k−m,α);(−λαt),k=0,1,….

The governing difference-differential equation is given by

(27) $$\frac{d}{dt}P^{\alpha ,\mu }\left(k,t\right)=-\left({\left(\mu +\lambda \left(1-B\right)\right)}^{\alpha }-\mu^{\alpha }\right)P^{\alpha ,\mu }\left(k,t\right),\;\;k>0.$$

The characteristic function of TSFPP,

(28) $$E\left[e^{iuN_{\alpha ,\mu }\left(t\right)}\right]=e^{-t({\left(\mu +\lambda \left(1-e^{iu}\right)\right)}^{\alpha }-\mu^{\alpha })}.$$

While using a standard conditioning argument, the mean and variance of TSFPP are given by

(29) $$\begin{array}{c} E\left[N_{\alpha ,\mu }\left(t\right)\right]=\lambda \alpha \mu^{\alpha -1}t,\;\;\mathrm{Var}\left[N_{\alpha ,\mu }\left(t\right)\right)]=\lambda \alpha \mu^{\alpha -1}t+\lambda^{2}\alpha \left(1-\alpha \right)\mu^{\alpha -2}t. \end{array}$$

**Proposition** **2.** *The Lévy density $\nu_{N_{\alpha ,\mu }}\left(x\right)$ of TSFPP is*

(30) νNα,μ(x)=∑n=1∞μα−nαnλn∑l=1nnl(−1)l+1δl(x),μ>0.

**Proof.**  Using (28), the characteristic exponent of TSFPP is given by $\psi_{N_{\alpha ,\mu }}\left(u\right)=-\left({\left(\mu +\lambda \left(1-e^{iu}\right)\right)}^{\alpha }-\mu^{\alpha }\right)$. We find the Lévy density with the help of Lévy-Khintchine formula (see [20]),

∫R\{0}(eiux−1)∑n=1∞μα−nαnλn∑l=1nnl(−1)l+1δl(x)dx=∑n=1∞μα−nαnλn∑l=1nnl(−1)l+1eiux−∑l=1nnl(−1)l+1=∑n=0∞μα−nαnλn∑l=0nnl(−1)l+1δl(x)−μα=−((μ+λ(1−eiu))α−μα),

hence proved. □

**Definition** **1.** *A stochastic process X(t) is over-dispersed, equi-dispersed or under-dispersed [18], if the Fisher index of dispersion, given by (see e.g., [17])*

$$\mathrm{FI}\left[X\left(t\right)\right]=\frac{\mathrm{Var}[X(t)]}{E[X(t)]}$$

*is more than 1, equal to 1, or smaller than 1, respectively, for all t>0.*

**Remark** **1.** *Using (29), we have $\mathrm{FI}\left[N_{\alpha ,\mu }\left(t\right)\right]=1+\frac{\lambda (1-\alpha )}{\mu }>1,$ i.e. TSFPP $N_{\alpha ,\mu }\left(t\right)$ is over-dispersed.*

## 3. Running Average of PPoK

In this section, we first introduced the running average of PPoK and their main properties. These results will be used further to discuss the running average of SPoK.

**Definition** **2** (Running average of PPoK). *We define the running average process $N_{A}^{k}\left(t\right),\;t\geq 0$ by taking time-scaled integral of the path of the PPoK (see [26]),*

(31) $$\begin{array}{c} N_{A}^{k}\left(t\right)=\frac{1}{t}\int_{0}^{t}N^{k}\left(s\right)ds. \end{array}$$

We can write the differential equation with initial condition $N_{A}^{k}\left(0\right)=0$,

$$\frac{d}{dt}\left(N_{A}^{k}\left(t\right)\right)=\frac{1}{t}N^{k}\left(t\right)-\frac{1}{t^{2}}\int_{0}^{t}N^{k}\left(s\right)ds.$$

Which shows that it has continuous sample paths of bounded total variation. We explored the compound Poisson representation and distribution properties of running average of PPoK. The characteristic of $N_{A}^{k}\left(t\right)$ is obtained using the Lemma 1 of [26]. We recall Lemma 1 from [26] for ease of reference.

**Lemma** **1.** *If $X_{t}$ is a Lévy process and $Y_{t}$ its Riemann integral is defined by*

$$\begin{array}{c} Y_{t}=\int_{0}^{t}X_{s}ds, \end{array}$$

*then the characteristic functions of Y satisfies*

(32) $$\phi_{Y(t)}\left(u\right)=E\left[e^{iuY(t)}\right]=e^{t\left(\int_{0}^{1}\log\phi_{X(1)}\left(tuz\right)dz\right)},\;u\in R.$$

**Proposition** **3.** *The characteristic function of $N_{A}^{k}\left(t\right)$ is given by*

(33) $$\phi_{N_{A}^{k}\left(t\right)}\left(u\right)=e^{-t\lambda \left(k-\sum_{j=1}^{k}\frac{\left(e^{iuj}-1\right)}{iuj}\right)}.$$

**Proof.**  Using the Equation (10), we have

$$\int_{0}^{1}\log\phi_{N^{k}\left(1\right)}\left(tuz\right)dz=-\lambda \left(k-\sum_{j=1}^{k}\frac{\left(e^{ituzj}-1\right)}{ituj}\right).$$

Using (32) and (31), we have

$$\begin{array}{c} \phi_{N_{A}^{k}\left(t\right)}\left(u\right)=e^{t\left(\int_{0}^{1}\log\phi_{N^{k}\left(1\right)}\left(uz\right)dz\right)}=e^{-t\lambda \left(k-\sum_{j=1}^{k}\frac{\left(e^{iuj}-1\right)}{iuj}\right)}. \end{array}$$

**Proposition** **4.** *The running average process has a compound Poisson representation, such that*

(34) $$\begin{array}{c} Y\left(t\right)=\sum_{i=1}^{N(t)}X_{i}, \end{array}$$

*where $X_{i}=1,2,\ldots$ are independent, identically distributed (iid) copies of X random variables, independent of N(t) and N(t) is a Poisson process with intensity kλ. Subsequently,*

$$Y\left(t\right)\overset{law}{=}N_{A}^{k}\left(t\right).$$

*Further, the random variable X has following pdf*

(35) $$f_{X}\left(x\right)=\sum_{i=1}^{k}p_{V_{i}}\left(x\right)f_{U_{i}}\left(x\right)=\frac{1}{k}\sum_{i=1}^{k}f_{U_{i}}\left(x\right),$$

*where $V_{i}$ follows discrete uniform distribution over (0,k) and $U_{i}$ follows continuous uniform distribution over (0,i),i=1,2,…,k.*

**Proof.**  The pdf of $U_{i}$ is $f_{U_{i}}\left(x\right)=\frac{1}{i},\;0\leq x\leq i.$ Using (35), the characteristic function of *X* is given by

$$\phi_{X}\left(u\right)=\frac{1}{k}\sum_{j=1}^{k}\frac{\left(e^{iuj}-1\right)}{iuj}.$$

For fixed *t*, the characteristic function of Y(t) is

(36) $$\phi_{Y(t)}\left(u\right)=e^{-k\lambda t(1-\phi_{X}\left(u\right))}=e^{-t\lambda \left(k-\sum_{j=1}^{k}\frac{\left(e^{iuj}-1\right)}{iuj}\right)},$$

which is equal to the characteristic function of PPoK that is given in (33). Hence, by the uniqueness of characteristic function, the result follows. □

Using the definition

(37) $$m_{r}=E\left[X^{r}\right]={\left(-i\right)}^{r}\frac{d^{r}\phi_{X}\left(u\right)}{du^{r}},$$

the first two moments for random variable *X* given in Proposition (4) are $m_{1}=\frac{\left(k+1\right)}{4}$ and $m_{2}=\frac{1}{18}\left[\left(k+1\right)\left(2k+1\right)\right]$. Further, using the mean, variance, and covariance of compound Poisson process, we have

$$\begin{array}{cc} E[N_{A}^{k}\left(t\right)] & =E\left[N\left(t\right)\right]E\left[X\right]=\frac{k(k+1)}{4}\lambda t;\\ \mathrm{Var}[N_{A}^{k}\left(t\right)] & =E\left[N\left(t\right)\right]E\left[X^{2}\right]=\frac{1}{18}\left[k\left(k+1\right)\left(2k+1\right)\right]\lambda t;\\ \mathrm{Cov}[N_{A}^{k}\left(t\right),N_{A}^{k}\left(s\right)] & =E\left[N_{A}^{k}\left(t\right),N_{A}^{k}\left(s\right)\right]-E\left[N_{A}^{k}\left(t\right)\right]E\left[N_{A}^{k}\left(s\right)\right]\\ \; & =E\left[N_{A}^{k}\left(s\right)\right]E\left[N_{A}^{k}\left(t-s\right)\right]-E\left[N_{A}^{k}{\left(s\right)}^{2}\right]-E\left[N_{A}^{k}\left(t\right)\right]E\left[N_{A}^{k}\left(s\right)\right]\\ \; & =\frac{1}{18}\left[k\left(k+1\right)\left(2k+1\right)\right]\lambda s-\frac{k^{2}{\left(k+1\right)}^{2}}{16}\lambda^{2}s^{2},\;s<t. \end{array}$$

**Corollary** **1.** *Putting k=1, the running average of PPoK $N_{A}^{k}\left(t\right)$ reduces to the running average of standard Poisson process $N_{A}\left(t\right)$ (see Appendix in [26]).*

**Corollary** **2.** *The mean and variance of PPoK and running average of PPoK satisfy, $E\left[N_{A}^{k}\left(t\right)\right]/E\left[N^{k}\left(t\right)\right]=\frac{1}{2}$ and $\mathrm{Var}\left[N_{A}^{k}\left(t\right)\right]/\mathrm{Var}\left[N^{k}\left(t\right)\right]=\frac{1}{3}$.*

**Remark** **2.** *The Fisher index of dispersion for running average of PPoK $N_{A}^{k}\left(t\right)$ is given by $\mathrm{FI}\left[N_{A}^{k}\left(t\right)\right]=\frac{2}{9}\left(2k+1\right).$ If k=1 the process is under-dispersed and for k>1 it is over-dispersed.*

Next we discuss the long-range dependence (LRD) property of running average of PPoK. We recall the definition of LRD for a non-stationary process.

**Definition** **3**  (Long range dependence (LRD)). *Let X(t) be a stochastic process that has a correlation function for st for fixed s, that satisfies,*

$$c_{1}\left(s\right)t^{-d}\leq \mathrm{Cor}\left(X\left(t\right),X\left(s\right)\right)\leq c_{2}\left(s\right)t^{-d},$$

*for large t, d>0, $c_{1}\left(s\right)>0$ and $c_{2}\left(s\right)>0$. For the particular case when $c_{1}\left(s\right)=c_{2}\left(s\right)=c\left(s\right)$, the above equation reduced to*

$$\lim_{t\to \infty }\frac{\mathrm{Cor}(X(t),X(s))}{t^{-d}}=c\left(s\right).$$

*We say that, if d∈(0,1), then X(t) has the LRD property and if d∈(1,2) it has short-range dependence (SRD) property [27].*

**Proposition** **5.** *The running average of PPoK has LRD property.*

**Proof.**  Let 0≤s<t<∞, then the correlation function for running average of PPoK $N_{A}^{k}\left(t\right)$ is

$$\begin{array}{cc} \mathrm{Cor}[N_{A}^{k}\left(t\right),N_{A}^{k}\left(s\right)] & =\frac{\left(8(2k+1)-9(k+1)k\lambda s\right)s^{1/2}t^{-1/2}}{8(2k+1)}. \end{array}$$

Subsequently, for d=1/2, it follows

$$\begin{array}{cc} \lim_{t\to \infty }\frac{\mathrm{Cor}[N_{A}^{k}\left(t\right),N_{A}^{k}\left(s\right)]}{t^{-d}} & =\frac{\left(8(2k+1)-9(k+1)k\lambda s\right)s^{1/2}}{8(2k+1)}=c\left(s\right). \end{array}$$

□

## 4. Skellam Process of Order *k* (SPoK)

In this section, we introduce and study the Skellam process of order *k* (SPoK).

**Definition** **4** (SPoK). *Let $N_{1}^{k}\left(t\right)$ and $N_{2}^{k}\left(t\right)$ be two independent PPoK with intensities $\lambda_{1}>0$ and $\lambda_{2}>0$. The stochastic process*

$$S^{k}\left(t\right)=N_{1}^{k}\left(t\right)-N_{2}^{k}\left(t\right)$$

*is called a Skellam process of order k (SPoK).*

**Proposition** **6.** *The marginal distribution $R_{m}\left(t\right)=P\left(S^{k}\left(t\right)=m\right)$ of SPoK $S^{k}\left(t\right)$ is given by*

(38) $$R_{m}\left(t\right)=e^{-kt(\lambda_{1}+\lambda_{2})}{\left(\frac{\lambda_{1}}{\lambda_{2}}\right)}^{m/2}I_{\left|m\right|}\left(2tk\sqrt{\lambda_{1}\lambda_{2}}\right),\;m\in Z.$$

**Proof.**  For m≥0, using the pmf of PPoK that is given in (8), it follows

$$\begin{array}{cc} R_{m}\left(t\right) & =\sum_{n=0}^{\infty }P\left(N_{1}^{k}\left(t\right)=n+m\right)P\left(N_{2}^{k}\left(t\right)=n\right)I_{m\geq 0}\\ \; & =\sum_{n=0}^{\infty }\left(\sum_{X=\Omega (k,n+m)}e^{-k\lambda_{1}t}\frac{{\left(\lambda_{1}t\right)}^{\zeta_{k}}}{\Pi_{k}!}\right)\left(\sum_{X=\Omega (k,n)}e^{-k\lambda_{2}t}\frac{{\left(\lambda_{2}t\right)}^{\zeta_{k}}}{\Pi_{k}!}\right). \end{array}$$

Setting $x_{i}=n_{i}$ and $n=x+\sum_{i=1}^{k}\left(i-1\right)n_{i}$, we have

Rm(t)=e−kt(λ1+λ2)∑x=0∞(λ2t)xx!(λ1t)m+x(m+x)!∑n1+n2+…+nk=m+xm+xn1!n2!…nk!∑n1+n2+…+nk=xxn1!n2!…nk!=e−kt(λ1+λ2)∑x=0∞(λ2t)xx!(λ1t)m+x(m+x)!km+xkx,

using the multinomial theorem and modified Bessel function given in (2). Similarly, it follows for m<0. □

**Proposition** **7.** *The Lévy density for SPoK is*

$$\nu_{S^{k}}\left(x\right)=\lambda_{1}\sum_{j=1}^{k}\delta_{j}\left(x\right)+\lambda_{2}\sum_{j=1}^{k}\delta_{-j}\left(x\right).$$

**Proof.**  The proof follows by using the independence of two PPoK used in the definition of SPoK. □

**Remark** **3.** *Using (12), the pgf of SPoK is given by*

(39) $$G^{S^{k}}\left(s,t\right)=\sum_{m=-\infty }^{\infty }s^{m}R_{m}\left(t\right)=e^{-t\left(k\left(\lambda_{1}+\lambda_{2}\right)-\lambda_{1}\sum_{j=1}^{k}s^{j}-\lambda_{2}\sum_{j=1}^{k}s^{-j}\right)}.$$

*Further, the characteristic function of SPoK is given by*

(40) $$\phi_{S^{k}\left(t\right)}\left(u\right)=e^{-t[k\left(\lambda_{1}+\lambda_{2}\right)-\lambda_{1}\sum_{j=1}^{k}e^{iju}-\lambda_{2}\sum_{j=1}^{k}e^{-iju}]}.$$

### SPoK as a Pure Birth and Death Process

In this section, we provide the transition probabilities of SPoK at time t+δ, given that we started at time *t*. Over such a short interval of length δ→0, it is nearly impossible to observe more than *k* event; in fact, the probability to see more than *k* event is o(δ).

**Proposition** **8.** *The transition probabilities of SPoK are given by*

(41) $$P\left(S^{k}\left(t+\delta \right)=m|S^{k}\left(t\right)=n\right)=\left\{\begin{array}{cc} \lambda_{1}\delta +o\left(\delta \right), & m>n,\;m=n+i,i=1,2,\ldots ,k;\\ \lambda_{2}\delta +o\left(\delta \right), & m<n,\;m=n-i,i=1,2,\ldots ,k;\\ 1-k\lambda_{1}\delta -k\lambda_{2}\delta +o\left(\delta \right), & m=n;\\ o\left(\delta \right) & \mathrm{otherwise}. \end{array}\right.$$

*Basically, at most k events can occur in a very small interval of time δ. Additionally, even though the probability for more than k event is non-zero, it is negligible.*

**Proof.**  Note that $S^{k}\left(t\right)=N_{1}^{k}\left(t\right)-N_{2}^{k}\left(t\right)$. We call $N_{1}^{k}\left(t\right)$ as the first process and $N_{2}^{k}\left(t\right)$ as the second process. For i=1,2,⋯,k, we have

$$\begin{array}{cc} P(S^{k}\left(t+\delta \right)=n+i|S^{k}\left(t\right)=n) & =\sum_{j=1}^{k-i}P\left(\mathrm{the}\mathrm{first}\mathrm{process}\mathrm{has}i+j\mathrm{arrivals}\mathrm{and}\mathrm{the}\mathrm{second}\mathrm{process}\mathrm{has}j\mathrm{arrivals}\right)\\ \; & +P(\mathrm{the}\mathrm{first}\mathrm{process}\mathrm{has}i\mathrm{arrivals}\mathrm{and}\mathrm{the}\mathrm{second}\mathrm{process}\mathrm{has}0\mathrm{arrivals})+o(\delta )\\ \; & =\sum_{j=0}^{k-i}\left(\lambda_{1}\delta +o\left(\delta \right)\right)\times \left(\lambda_{2}\delta +o\left(\delta \right)\right)+\left(\lambda_{1}\delta +o\left(\delta \right)\right)\times \left(1-k\lambda_{2}\delta +o\left(\delta \right)\right)+o\left(\delta \right)\\ \; & =\lambda_{1}\delta +o\left(\delta \right). \end{array}$$

Similarly, for i=1,2,⋯,k, we have

$$\begin{array}{cc} P(S^{k}\left(t+\delta \right)=n-i|S^{k}\left(t\right)=n) & =\sum_{j=1}^{k-i}P\left(\mathrm{the}\mathrm{first}\mathrm{process}\mathrm{has}j\mathrm{arrivals}\mathrm{and}\mathrm{the}\mathrm{second}\mathrm{process}\mathrm{has}i+j\mathrm{arrivals}\right)\\ \; & +P(\mathrm{the}\mathrm{first}\mathrm{process}\mathrm{has}0\mathrm{arrivals}\mathrm{and}\mathrm{the}\mathrm{second}\mathrm{process}\mathrm{has}i\mathrm{arrivals})+o(\delta )\\ \; & =\sum_{j=0}^{k-i}\left(\lambda_{1}\delta +o\left(\delta \right)\right)\times \left(\lambda_{2}\delta +o\left(\delta \right)\right)+\left(1-k\lambda_{1}\delta +o\left(\delta \right)\right)\times \left(\lambda_{2}\delta +o\left(\delta \right)\right)+o\left(\delta \right)\\ \; & =\lambda_{2}\delta +o\left(\delta \right). \end{array}$$

Further,

$$\begin{array}{cc} P(S^{k}\left(t+\delta \right)=n|S^{k}\left(t\right)=n) & =\sum_{j=1}^{k}P\left(\mathrm{the}\mathrm{first}\mathrm{process}\mathrm{has}j\mathrm{arrivals}\mathrm{and}\mathrm{the}\mathrm{second}\mathrm{process}\mathrm{has}j\mathrm{arrivals}\right)\\ \; & +P(\mathrm{the}\mathrm{first}\mathrm{process}\mathrm{has}0\mathrm{arrivals}\mathrm{and}\mathrm{the}\mathrm{second}\mathrm{process}\mathrm{has}0\mathrm{arrivals})+o(\delta )\\ \; & =\sum_{j=0}^{k}\left(\lambda_{1}\delta +o\left(\delta \right)\right)\times \left(\lambda_{2}\delta +o\left(\delta \right)\right)+\left(1-k\lambda_{1}\delta +o\left(\delta \right)\right)\times \left(1-k\lambda_{2}\delta +o\left(\delta \right)\right)+o\left(\delta \right)\\ \; & =1-k\lambda_{1}\delta -k\lambda_{2}\delta +o\left(\delta \right). \end{array}$$

□

**Remark** **4.** *The pmf $R_{m}\left(t\right)$ of SPoK satisfies the following difference differential equation*

$$\begin{array}{cc} \frac{d}{dt}R_{m}\left(t\right) & =-k\left(\lambda_{1}+\lambda_{2}\right)R_{m}\left(t\right)+\lambda_{1}\sum_{j=1}^{k}R_{m-j}\left(t\right)+\lambda_{2}\sum_{j=1}^{k}R_{m+j}\left(t\right)\\ \; & =-\lambda_{1}\sum_{j=1}^{k}\left(1-B^{j}\right)R_{m}-\lambda_{2}\sum_{j=1}^{k}\left(1-F^{j}\right)R_{m}\left(t\right),\;\;m\in Z, \end{array}$$

*with initial condition $R_{0}\left(0\right)=1$ and $R_{m}\left(0\right)=0$ for m≠0. Let B be the backward shift operator defined in (22) and F be the forward shift operator defined by $F^{j}X\left(t\right)=X\left(t+j\right)$, such that (1−F)α=∑j=0∞αjFj. Multiplying by $s^{m}$ and summing for all m in (42), we obtain the following differential equation for the pgf*

$$\frac{d}{dt}G^{S^{k}}\left(s,t\right)=\left(-k\left(\lambda_{1}+\lambda_{2}\right)+\lambda_{1}\sum_{j=1}^{k}s^{j}+\lambda_{2}\sum_{j=1}^{k}s^{-j}\right)G^{S^{k}}\left(s,t\right).$$

The mean, variance and covariance of SPoK can be easily calculated by using the pgf,

$$\begin{array}{cc} E[S^{k}\left(t\right)] & =\frac{k(k+1)}{2}\left(\lambda_{1}-\lambda_{2}\right)t;\\ \mathrm{Var}[S^{k}\left(t\right)] & =\frac{1}{6}\left[k(k+1)(2k+1)\right]\left(\lambda_{1}+\lambda_{2}\right)t;\\ \mathrm{Cov}[S^{k}\left(t\right),S^{k}\left(s\right)] & =\frac{1}{6}\left[k(k+1)(2k+1)\right]\left(\lambda_{1}+\lambda_{2}\right)s,\;\;s<t. \end{array}$$

**Remark** **5.** *For the SPoK, when $\lambda_{1}>\lambda_{2}$, $\mathrm{Var}\left[S^{k}\left(t\right)\right]-E\left[S^{k}\left(t\right)\right]=\frac{k(k+1)}{3}[\left(k-1\right)\lambda_{1}+\left(k+2\right)\lambda_{2}>0,$ which implies that $\mathrm{FI}[S^{k}\left(t\right)]>1$ and hence $S^{k}\left(t\right)$ exhibits over-dispersion. For $\lambda_{1}<\lambda_{2}$, the process is under-dispersed.*

Next, we show the LRD property for SPoK.

**Proposition** **9.** *The SPoK has LRD property defined in Definition 3.*

**Proof.**  The correlation function of SPoK satisfies

$$\lim_{t\to \infty }\frac{\mathrm{Cor}(S^{k}\left(t\right),S^{k}\left(s\right))}{t^{-d}}=\frac{s^{1/2}t^{-1/2}}{t^{-1/2}}=c\left(s\right).$$

Hence, SPoK exhibits the LRD property. □

## 5. Running Average of SPoK

In this section, we introduce and study the new stochastic Lévy process, which is the running average of SPoK.

**Definition** **5.** *The following stochastic process defined by taking the time-scaled integral of the path of the SPoK,*

(42) $$\begin{array}{c} S_{A}^{k}\left(t\right)=\frac{1}{t}\int_{0}^{t}S^{k}\left(s\right)ds, \end{array}$$

*is called the running average of SPoK.*

Next, we provide the compound Poisson representation of running average of SPoK.

**Proposition** **10.** *The characteristic function $\phi_{S_{A}^{k}\left(t\right)}\left(u\right)=E\left[e^{iuS_{A}^{k}\left(t\right)}\right]$ of $S_{A}^{k}\left(t\right)$ is given by*

(43) $$\begin{array}{c} \phi_{S_{A}^{k}\left(t\right)}\left(u\right)=e^{-kt\left\{\lambda_{1}\left(1-\frac{1}{k}\sum_{j=1}^{k}\frac{\left(e^{iuj}-1\right)}{iuj}\right)+\lambda_{2}\left(1-\frac{1}{k}\sum_{j=1}^{k}\frac{\left(1-e^{-iuj}\right)}{iuj}\right)\right\}},\;u\in R. \end{array}$$

**Proof.**  By using the Lemma 3.1 to Equation (40) after scaling by 1/t. □

**Remark** **6.** *It is easily observable that Equation (43) has removable singularity at u=0. To remove that singularity, we can define $\phi_{S_{A}^{k}\left(t\right)}\left(0\right)=1$.*

**Proposition** **11.** *Let Y(t) be a compound Poisson process*

(44) $$\begin{array}{c} Y\left(t\right)=\sum_{n=1}^{N(t)}J_{n}, \end{array}$$

*where N(t) is a Poisson process with rate parameter $k(\lambda_{1}+\lambda_{2})>0$ and ${\left\{J_{n}\right\}}_{n\geq 1}$ are iid random variables with mixed double uniform distribution function $p_{j}$, which are independent of N(t). Subsequently,*

$$Y\left(t\right)\overset{law}{=}S_{A}^{k}\left(t\right).$$

**Proof.**  Rearranging the $\phi_{S_{A}^{k}\left(t\right)}\left(u\right)$,

$$\phi_{S_{A}^{k}\left(t\right)}\left(u\right)=e^{\left(\lambda_{1}+\lambda_{2}\right)kt\left(\frac{\lambda_{1}}{\lambda_{1}+\lambda_{2}}\frac{1}{k}\sum_{j=1}^{k}\frac{\left(e^{iuj}-1\right)}{iuj}+\frac{\lambda_{2}}{\lambda_{1}+\lambda_{2}}\frac{1}{k}\sum_{j=1}^{k}\frac{\left(1-e^{-iuj}\right)}{iuj}-1\right)}$$

The random variable $J_{1}$ being a mixed double uniformly distributed has density

(45) $$p_{J_{1}}\left(x\right)=\sum_{i=1}^{k}p_{V_{i}}\left(x\right)f_{U_{i}}\left(x\right)=\frac{1}{k}\sum_{i=1}^{k}f_{U_{i}}\left(x\right),$$

where $V_{j}$ follows discrete uniform distribution over (0,k) with pmf $p_{V_{j}}\left(x\right)=P\left(V_{j}=x\right)=\frac{1}{k},\;\;j=1,2,\ldots k,$ and $U_{i}$ be doubly uniform distributed random variables with density

$$f_{U_{i}}\left(x\right)=\left(1-w\right)1_{\left[-i,0\right]}\left(x\right)+w1_{\left[0,i\right]}\left(x\right),\;\;-i\leq x\leq i.$$

Further, 0<w<1 is a weight parameter and 1(·) is the indicator function. Here, we obtained the characteristic of $J_{1}$ using the Fourier transform of (45),

$$\phi_{J_{1}}\left(u\right)=\frac{\lambda_{1}}{\lambda_{1}+\lambda_{2}}\frac{1}{k}\sum_{j=1}^{k}\frac{\left(e^{iuj}-1\right)}{iuj}+\frac{\lambda_{2}}{\lambda_{1}+\lambda_{2}}\frac{1}{k}\sum_{j=1}^{k}\frac{\left(1-e^{-iuj}\right)}{iuj}.$$

The characteristic function of Y(t) is

(46) $$\phi_{Y(t)}\left(u\right)=e^{-kt\left(\lambda_{1}+\lambda_{2}\right)t\left(1-\phi_{J_{1}}\left(u\right)\right)},$$

putting the characteristic function $\phi_{J_{1}}\left(u\right)$ in the above expression yields the characteristic function of $S_{A}^{k}\left(t\right)$, which completes the proof. □

**Remark** **7.** *The q-th order moments of $J_{1}$ can be calculated using (37) and also using Taylor series expansion of the characteristic $\phi_{J_{1}}\left(u\right)$, around 0, such that*

$$\frac{\left(e^{iuj}-1\right)}{iuj}=1+\sum_{r=1}^{\infty }\frac{{\left(iuj\right)}^{r}}{(r+1)!}\;\;\&\;\;\frac{\left(1-e^{-iuj}\right)}{iuj}=1+\sum_{r=1}^{\infty }\frac{{\left(-iuj\right)}^{r}}{(r+1)!}.$$

*We have $m_{1}=\frac{\left(k+1\right)\left(\lambda_{1}-\lambda_{2}\right)}{4(\lambda_{1}+\lambda_{2})}$ and $m_{2}=\frac{1}{18}\left[\left(k+1\right)\left(2k+1\right)\right]$. Further, the mean, variance, and covariance of running average of SPoK are*

$$\begin{array}{cc} E[S_{A}^{k}\left(t\right)] & =E\left[N\left(t\right)\right]E\left[J_{1}\right]=\frac{k(k+1)}{4}\left(\lambda_{1}-\lambda_{2}\right)t\\ \mathrm{Var}[S_{A}^{k}\left(t\right)] & =E\left[N\left(t\right)\right]E\left[J_{1}^{2}\right]=\frac{1}{18}\left[k\left(k+1\right)\left(2k+1\right)\right]\left(\lambda_{1}+\lambda_{2}\right)t\\ \mathrm{Cov}[S_{A}^{k}\left(t\right),S_{A}^{k}\left(s\right)] & =\frac{1}{18}\left[k\left(k+1\right)\left(2k+1\right)\right]\left(\lambda_{1}-\lambda_{2}\right)s-\frac{k^{2}{\left(k+1\right)}^{2}}{16}{\left(\lambda_{1}-\lambda_{2}\right)}^{2}s^{2}. \end{array}$$

**Corollary** **3.** *For $\lambda_{2}=0$ the running average of SPoK is same as the running average of PPoK, i.e.,*

$$\phi_{S_{A}^{k}\left(t\right)}\left(u\right)=\phi_{N_{A}^{k}\left(t\right)}\left(u\right).$$

**Corollary** **4.** *For k=1 this process behave like the running average of Skellam process.*

**Corollary** **5.** *The ratio of mean and variance of SPoK and running average of SPoK are 1/2 and 1/3, respectively.*

**Remark** **8.** *For running average of SPoK, when $\lambda_{1}>\lambda_{2}$ and k>1, the process is over-dispersed. Otherwise, it exhibits under-dispersion.*

## 6. Time-Changed Skellam Process of Order *k*

We consider time-changed SPoK, which can be obtained by subordinating SPoK $S^{k}\left(t\right)$ with the independent Lévy subordinator $D_{f}\left(t\right)$ satisfying $E{\left[D_{f}\left(t\right)\right]}^{c}<\infty$ for all c>0. The time-changed SPoK is defined by

$$Z_{f}\left(t\right)=S^{k}\left(D_{f}\left(t\right)\right),\;\;t\geq 0.$$

Note that the stable subordinator does not satisfy the condition $E{\left[D_{f}\left(t\right)\right]}^{c}<\infty$. The mgf of time-changed SPoK $Z_{f}\left(t\right)$ is given by

$$E\left[e^{\theta Z_{f}\left(t\right)}\right]=e^{-tf(k\left(\lambda_{1}+\lambda_{2}\right)-\lambda_{1}\sum_{j=1}^{k}e^{\theta j}-\lambda_{2}\sum_{j=1}^{k}e^{-\theta j})}.$$

**Theorem** **2.** *The pmf $H_{f}\left(t\right)=P\left(Z_{f}\left(t\right)=m\right)$ of time-changed SPoK is given by*

(47) $$H_{f}\left(t\right)=\sum_{x=\max(0,-m)}^{\infty }\frac{{\left(k\lambda_{1}\right)}^{m+x}{\left(k\lambda_{2}\right)}^{x}}{(m+x)!x!}E\left[e^{-k\left(\lambda_{1}+\lambda_{2}\right)D_{f}\left(t\right)}D_{f}^{2m+x}\left(t\right)\right],\;m\in Z.$$

**Proof.**  Let $h_{f}\left(x,t\right)$ be the probability density function of Lévy subordinator. Using conditional argument

$$\begin{array}{cc} H_{f}\left(t\right) & =\int_{0}^{\infty }R_{m}\left(y\right)h_{f}\left(y,t\right)dy\\ \; & =\int_{0}^{\infty }e^{-ky(\lambda_{1}+\lambda_{2})}{\left(\frac{\lambda_{1}}{\lambda_{2}}\right)}^{m/2}I_{\left|m\right|}\left(2yk\sqrt{\lambda_{1}\lambda_{2}}\right)h_{f}\left(y,t\right)dy\\ \; & =\sum_{x=\max(0,-m)}^{\infty }\frac{{\left(k\lambda_{1}\right)}^{m+x}{\left(k\lambda_{2}\right)}^{x}}{(m+x)!x!}\int_{0}^{\infty }e^{-k(\lambda_{1}+\lambda_{2})y}y^{2m+x}h_{f}\left(y,t\right)dy\\ \; & =\sum_{x=\max(0,-m)}^{\infty }\frac{{\left(k\lambda_{1}\right)}^{m+x}{\left(k\lambda_{2}\right)}^{x}}{(m+x)!x!}E\left[e^{-k\left(\lambda_{1}+\lambda_{2}\right)D_{f}\left(t\right)}D_{f}^{2m+x}\left(t\right)\right]. \end{array}$$

□

The mean and covariance of time changed SPoK are given by,

$$\begin{array}{cc} E[Z_{f}\left(t\right)] & =\frac{k(k+1)}{2}\left(\lambda_{1}-\lambda_{2}\right)E\left[D_{f}\left(t\right)\right].\\ \mathrm{Cov}[Z_{f}\left(t\right),Z_{f}\left(s\right)] & =\frac{1}{6}\left[k\left(k+1\right)\left(2k+1\right)\right]\left(\lambda_{1}+\lambda_{2}\right))E\left[D_{f}\left(s\right)\right]+\frac{k^{2}{\left(k+1\right)}^{2}}{4}{\left(\lambda_{1}-\lambda_{2}\right)}^{2}\mathrm{Var}\left[D_{f}\left(s\right)\right]. \end{array}$$

## 7. Space Fractional Skellam Process and Tempered Space Fractional Skellam Process

In this section, we introduce time-changed Skellam processes where time-change are stable subordinator and tempered stable subordinator. These processes give the space-fractional version of the Skellam process similar to the time-fractional version of the Skellam process introduced in [10].

### 7.1. The Space-Fractional Skellam Process

In this section, we introduce space-fractional Skellam processes (SFSP). Further, for introduced processes, we study main results, such as state probabilities and governing difference-differential equations of marginal pmf.

**Definition** **6** (SFSP). *Let $N_{1}\left(t\right)$ and $N_{2}\left(t\right)$ be two independent homogeneous Poison processes with intensities $\lambda_{1}>0$ and $\lambda_{2}>0,$, respectively. Let $D_{\alpha_{1}}\left(t\right)$ and $D_{\alpha_{2}}\left(t\right)$ be two independent stable subordinators with indices $\alpha_{1}\in \left(0,1\right)$ and $\alpha_{2}\in \left(0,1\right)$, respectively. These subordinators are independent of the Poisson processes $N_{1}\left(t\right)$ and $N_{2}\left(t\right)$. The subordinated stochastic process*

$$S_{\alpha_{1},\alpha_{2}}\left(t\right)=N_{1}\left(D_{\alpha_{1}}\left(t\right)\right)-N_{2}\left(D_{\alpha_{2}}\left(t\right)\right)$$

*is called a SFSP.*

Next, we derive the mgf of SFSP. We use the expression for marginal (pmf) of SFPP that is given in (17) to obtain the marginal pmf of SFSP.

$$\begin{array}{cc} M_{\theta }\left(t\right)=E\left[e^{\theta S_{\alpha_{1},\alpha_{2}}\left(t\right)}\right] & =E\left[e^{\theta (N_{1}\left(D_{\alpha_{1}}\left(t\right)\right)-N_{2}\left(D_{\alpha_{2}}\left(t\right)\right))}\right]=e^{-t[\lambda_{1}^{\alpha_{1}}{\left(1-e^{\theta }\right)}^{\alpha_{1}}+\lambda_{2}^{\alpha_{2}}{\left(1-e^{-\theta }\right)}^{\alpha_{2}}]},\;\;\theta \in R. \end{array}$$

In the next result, we obtain the state probabilities of the SFSP.

**Theorem** **3.** *The pmf $H_{k}\left(t\right)=P\left(S_{\alpha_{1},\alpha_{2}}\left(t\right)=k\right)$ of SFSP is given by*

(48) $$\begin{array}{cc} H_{k}\left(t\right) & =\sum_{n=0}^{\infty }\frac{{\left(-1\right)}^{k}}{n!(n+k)!}\left({}_{1}\psi_{1}\left[\begin{array}{c} (1,\alpha_{1});\\ (1-n-k,\alpha_{1}); \end{array}\left(-{\lambda_{1}}^{\alpha_{1}}t\right)\right]\right)\left({}_{1}\psi_{1}\left[\begin{array}{c} (1,\alpha_{2});\\ (1-n,\alpha_{2}); \end{array}\left(-{\lambda_{2}}^{\alpha_{2}}t\right)\right]\right)I_{k\geq 0}\\ \; & +\sum_{n=0}^{\infty }\frac{{\left(-1\right)}^{\left|k\right|}}{n!(n+|k|)!}\left({}_{1}\psi_{1}\left[\begin{array}{c} (1,\alpha_{1});\\ (1-n,\alpha_{1}); \end{array}\left(-{\lambda_{1}}^{\alpha_{1}}t\right)\right]\right)\left({}_{1}\psi_{1}\left[\begin{array}{c} (1,\alpha_{2});\\ (1-n-|k|,\alpha_{2}); \end{array}\left(-{\lambda_{2}}^{\alpha_{2}}t\right)\right]\right)I_{k<0} \end{array}$$

*for k∈Z.*

**Proof.**  Note that $N_{1}\left(D_{\alpha_{1}}\left(t\right)\right)$ and $N_{2}\left(D_{\alpha_{2}}\left(t\right)\right)$ are independent, hence

$$\begin{array}{cc} P(S_{\alpha_{1},\alpha_{2}}\left(t\right)=k) & =\sum_{n=0}^{\infty }P\left(N_{1}\left(D_{\alpha_{1}}\left(t\right)\right)=n+k\right)P\left(N_{2}\left(D_{\alpha_{2}}\left(t\right)\right)=n\right)I_{k\geq 0}\\ \; & +\sum_{n=0}^{\infty }P\left(N_{1}\left(D_{\alpha_{1}}\left(t\right)\right)=n\right)P(N_{2}\left(D_{\alpha_{2}}\left(t\right)\right)=n+\left|k|\right)I_{k<0}. \end{array}$$

Using (17), the result follows. □

In the next theorem, we discuss the governing differential-difference equation of the marginal pmf of SFSP.

**Theorem** **4.** *The marginal distribution $H_{k}\left(t\right)=P\left(S_{\alpha_{1},\alpha_{2}}\left(t\right)=k\right)$ of SFSP satisfies the following differential difference equations*

(49) $$\begin{array}{cc} \frac{d}{dt}H_{k}\left(t\right) & =-\lambda_{1}^{\alpha_{1}}{\left(1-B\right)}^{\alpha_{1}}H_{k}\left(t\right)-\lambda_{2}^{\alpha_{2}}{\left(1-F\right)}^{\alpha_{2}}H_{k}\left(t\right),\;\;k\in Z \end{array}$$

(50) $$\begin{array}{cc} \frac{d}{dt}H_{0}\left(t\right) & =-\lambda_{1}^{\alpha_{1}}H_{0}\left(t\right)-\lambda_{2}^{\alpha_{2}}H_{1}\left(t\right), \end{array}$$

*with initial conditions $H_{0}\left(0\right)=1$ and $H_{k}\left(0\right)=0$ for k≠0.*

**Proof.**  The proof follows by using pgf. □

**Remark** **9.** *The mgf of the SFSP solves the differential equation*

(51) $$\begin{array}{c} \frac{dM_{\theta }\left(t\right)}{dt}=-M_{\theta }\left(t\right)\left(\lambda_{1}^{\alpha_{1}}{\left(1-e^{\theta }\right)}^{\alpha_{1}}+\lambda_{2}^{\alpha_{2}}{\left(1-e^{-\theta }\right)}^{\alpha_{2}}\right). \end{array}$$

**Proposition** **12.** *The Lévy density $\nu_{S_{\alpha_{1},\alpha_{2}}}\left(x\right)$ of SFSP is given by*

νSα1,α2(x)=λ1α1∑n1=1∞(−1)n1+1α1n1δn1(x)+λ2α2∑n2=1∞(−1)n2+1α2n2δ−n2(x).

**Proof.**  Substituting the Lévy density $\nu_{N_{\alpha_{1}}}\left(x\right)$ and $\nu_{N_{\alpha_{2}}}\left(x\right)$ of $N_{1}\left(D_{\alpha_{1}}\left(t\right)\right)$ and $N_{2}\left(D_{\alpha_{2}}\left(t\right)\right)$, respectively, from the Equation (24), we obtain

$$\nu_{S_{\alpha_{1},\alpha_{2}}}\left(x\right)=\nu_{N_{\alpha_{1}}}\left(x\right)+\nu_{N_{\alpha_{2}}}\left(x\right),$$

which gives the desired result. □

### 7.2. Tempered Space-Fractional Skellam Process (TSFSP)

In this section, we present the tempered space-fractional Skellam process (TSFSP). We discuss the corresponding fractional difference-differential equations, marginal pmfs, and moments of this process.

**Definition** **7** (TSFSP). *The TSFSP is obtained by taking the difference of two independent tempered space fractional Poisson processes. Let $D_{\alpha_{1},\mu_{1}}\left(t\right)$, $D_{\alpha_{2},\mu_{2}}\left(t\right)$ be two independent TSS (see [28]) and $N_{1}\left(t\right),N_{2}\left(t\right)$ be two independent Poisson processes that are independent of TSS. Subsequently, the stochastic process*

$$S_{\alpha_{1},\alpha_{2}}^{\mu 1,\mu 2}\left(t\right)=N_{1}(D_{\alpha_{1},\mu_{1}}\left(t\right)-N_{2}\left(D_{\alpha_{2},\mu_{2}}\left(t\right)\right)$$

*is called the TSFSP.*

**Theorem** **5.** *The PMF $H_{k}^{\mu 1,\mu 2}\left(t\right)=P\left(S_{\alpha_{1},\alpha_{2}}^{\mu 1,\mu 2}\left(t\right)=k\right)$ is given by*

(52) $$\begin{array}{cc} H_{k}^{\mu 1,\mu 2}\left(t\right) & =\sum_{n=0}^{\infty }\frac{{\left(-1\right)}^{k}}{n!(n+k)!}e^{t(\mu_{1}^{\alpha_{1}}+\mu 1^{\alpha_{1}})}\left(\sum_{m=0}^{\infty }\frac{\mu_{1}^{m}\lambda_{1}^{-m}}{m!}{}_{1}\psi_{1}\left[\begin{array}{c} (1,\alpha_{1});\\ (1-n-k-m,\alpha_{1}); \end{array}\left(-{\lambda_{1}}^{\alpha_{1}}t\right)\right]\right)\times \\ \; & \left(\sum_{l=0}^{\infty }\frac{{\mu_{2}}^{l}{\lambda_{2}}^{-l}}{l!}{}_{1}\psi_{1}\left[\begin{array}{c} (1,\alpha_{2});\\ (1-l-k,\alpha_{2}); \end{array}\left(-{\lambda_{2}}^{\alpha_{2}}t\right)\right]\right) \end{array}$$

*when k≥0 and similarly for k<0,*

(53) $$\begin{array}{cc} H_{k}^{\mu 1,\mu 2}\left(t\right) & =\sum_{n=0}^{\infty }\frac{{\left(-1\right)}^{\left|k\right|}}{n!(n+|k|)!}e^{t(\mu_{1}^{\alpha_{1}}+\mu 1^{\alpha_{1}})}\left(\sum_{m=0}^{\infty }\frac{\mu_{1}^{m}\lambda_{1}^{-m}}{m!}{}_{1}\psi_{1}\left[\begin{array}{c} (1,\alpha_{1});\\ (1-n-m,\alpha_{1}); \end{array}\left(-{\lambda_{1}}^{\alpha_{1}}t\right)\right]\right)\times \\ \; & \left(\sum_{l=0}^{\infty }\frac{{\mu_{2}}^{l}{\lambda_{2}}^{-l}}{l!}{}_{1}\psi_{1}\left[\begin{array}{c} (1,\alpha_{2});\\ (1-l-n-|k|,\alpha_{2}); \end{array}\left(-{\lambda_{2}}^{\alpha_{2}}t\right)\right]\right). \end{array}$$

**Proof.**  Because $N_{1}\left(D_{\alpha_{1},\mu_{1}}\left(t\right)\right)$ and $N_{2}\left(D_{\alpha_{2},\mu_{2}}\left(t\right)\right)$ are independent,

$$\begin{array}{cc} P\left(S_{\alpha_{1},\alpha_{2}}^{\mu 1,\mu 2}\left(t\right)=k\right) & =\sum_{n=0}^{\infty }P\left(N_{1}\left(D_{\alpha_{1},\mu_{1}}\left(t\right)\right)=n+k\right)P\left(N_{2}\left(D_{\alpha_{2},\mu_{2}}\left(t\right)\right)=n\right)I_{k\geq 0}\\ \; & +\sum_{n=0}^{\infty }P\left(N_{1}\left(D_{\alpha_{1},\mu_{1}}\left(t\right)\right)=n\right)P(N_{2}\left(D_{\alpha_{2},\mu_{2}}\left(t\right)\right)=n+\left|k|\right)I_{k<0}, \end{array}$$

which gives the marginal pmf of TSFPP using (26). □

**Remark** **10.** *We use this expression to calculate the marginal distribution of TSFSP. The mgf is obtained using the conditioning argument. Let $f_{\alpha ,\mu }\left(x,t\right)$ be the density function of $D_{\alpha ,\mu }\left(t\right)$. Subsequently,*

(54) $$\begin{array}{cc} E[e^{\theta N(D_{\alpha ,\mu }\left(t\right))}] & =\int_{0}^{\infty }E\left[e^{\theta N(u)}\right]f_{\alpha ,\mu }\left(u,t\right)du=e^{-t\{{\left(\lambda \left(1-e^{\theta }\right)+\mu \right)}^{\alpha }-\mu^{\alpha }\}}. \end{array}$$

*Using (54), the mgf of TSFSP is*

$$E\left[e^{\theta S_{\alpha_{1},\alpha_{2}}^{\mu_{1},\mu_{2}}\left(t\right)}\right]=E\left[e^{\theta N_{1}\left(D_{\alpha_{1},\mu_{1}}\left(t\right)\right)}\right]E\left[e^{-\theta N_{2}\left(D_{\alpha_{2},\mu_{2}}\left(t\right)\right)}\right]=e^{-t[\left\{{\left(\lambda_{1}\left(1-e^{\theta }\right)+\mu_{1}\right)}^{\alpha_{1}}-\mu_{1}^{\alpha_{1}}\right\}+\left\{{\left(\lambda_{2}\left(1-e^{-\theta }\right)+\mu_{2}\right)}^{\alpha_{2}}-\mu_{2}^{\alpha_{2}}\right\}]}.$$

**Remark** **11.** *We have $E\left[S_{\alpha_{1},\alpha_{2}}^{\mu 1,\mu 2}\left(t\right)\right]=t\left(\lambda_{1}\alpha_{1}\mu_{1}^{\alpha_{1}-1}-\lambda_{2}\alpha_{2}\mu_{2}^{\alpha_{2}-1}\right).$ Further, the covariance of TSFSP can be obtained by using (29) and*

$$\begin{array}{cc} \mathrm{Cov}\left[S_{\alpha_{1},\alpha_{2}}^{\mu_{1},\mu_{2}}\left(t\right),S_{\alpha_{1},\alpha_{2}}^{\mu_{1},\mu_{2}}\left(s\right)\right] & =\mathrm{Cov}\left[N_{1}\left(D_{\alpha_{1},\mu_{1}}\left(t\right)\right),N_{1}\left(D_{\alpha_{1},\mu_{1}}\left(s\right)\right)\right]+\mathrm{Cov}\left[N_{2}\left(D_{\alpha_{2},\mu_{2}}\left(t\right)\right),N_{2}\left(D_{\alpha_{2},\mu_{2}}\left(s\right)\right)\right]\\ \; & =\mathrm{Var}(N_{1}\left(D_{\alpha_{1},\mu_{1}}\left(\min\left(t,s\right)\right)\right)+\mathrm{Var}(N_{2}\left(D_{\alpha_{2},\mu_{2}}\left(\min\left(t,s\right)\right)\right). \end{array}$$

**Proposition** **13.** *The Lévy density $\nu_{S_{\alpha_{1},\alpha_{2}}^{\mu_{1},\mu_{2}}}\left(x\right)$ of TSFSP is given by*

νSα1,α2μ1,μ2(x)=∑n1=1∞μ1α1−n1α1n1λ1n1∑l=1n1n1l1(−1)l1+1δl1(x)+∑n2=1∞μ2α2−n2α2n2λ2n2∑l2=1n2n2l2(−1)l2+1δl2(x),μ1,μ2>0.

**Proof.**  By adding Lévy density $\nu_{N_{\alpha_{1},\mu_{1}}}\left(x\right)$ and $\nu_{N_{\alpha_{2},\mu_{2}}}\left(x\right)$ of $N_{1}\left(D_{\alpha_{1},\mu_{1}}\left(t\right)\right)$ and $N_{2}\left(D_{\alpha_{2},\mu_{2}}\left(t\right)\right)$, respectively, from Equation (30), which leads to

$$\nu_{S_{\alpha_{1},\alpha_{2}}^{\mu 1,\mu 2}}\left(x\right)=\nu_{N_{\alpha_{1},\mu_{1}}}\left(x\right)+\nu_{N_{\alpha_{2},\mu_{2}}}\left(x\right).$$

□

### 7.3. Simulation of SFSP and TSFSP

We present the algorithm to simulate the sample trajectories for SFSP and TSFSP. We use *Python 3.7* and its libraries *Numpy* and *Matplotlib* for the simulation purpose. It is worth mentioning that Python is an open source and freely available software.

**Simulation of SFSP:** fix the values of the parameters $\alpha_{1}$, $\alpha_{2}$, $\lambda_{1}$ and $\lambda_{2};$

**Step-1:** generate independent and uniformly distributed random vectors *U*, *V* of size 1000 each in the interval [0,1];
**Step-2:** generate the increments of the $\alpha_{1}$-stable subordinator $D_{\alpha_{1}}\left(t\right)$ (see [29]) with pdf $f_{\alpha_{1}}\left(x,t\right)$, while using the relationship $D_{\alpha_{1}}\left(t+dt\right)-D_{\alpha_{1}}\left(t\right)\overset{d}{=}D_{\alpha_{1}}\left(dt\right)\overset{d}{=}{\left(dt\right)}^{\frac{1}{\alpha_{1}}}D_{\alpha_{1}}\left(1\right)$, where
  $$D_{\alpha_{1}}\left(1\right)=\frac{\sin\left(\alpha_{1}\pi U\right){\left[\sin\left(\left(1-\alpha_{1}\right)\pi U\right)\right]}^{1/\alpha_{1}-1}}{{\left[\sin\left(\pi U\right)\right]}^{1/\alpha_{1}}{\left|\log V\right|}^{1/\alpha_{1}-1}};$$
**Step-3:** generate the increments of Poisson distributed rvs $N_{1}\left(D_{\alpha_{1}}\left(dt\right)\right)$ with parameter $\lambda_{1}{\left(dt\right)}^{1/\alpha_{1}}D_{\alpha_{1}}\left(1\right)$;
**Step-4:** cumulative sum of increments gives the space fractional Poisson process $N_{1}\left(D_{\alpha_{1}}\left(t\right)\right)$ sample trajectories; and,
**Step-5:** similarly generate $N_{2}\left(D_{\alpha_{2}}\left(t\right)\right)$ and subtract these to obtain the SFSP $S_{\alpha_{1},\alpha_{2}}\left(t\right)$.

We next present the algorithm for generating the sample trajectories of TSFSP.

**Simulation of TSFSP:** fix the values of the parameters $\alpha_{1}$, $\alpha_{2}$, $\lambda_{1}$, $\lambda_{2}$, $\mu_{1}$ and $\mu_{2}.$

Use the first two steps of previous algorithm for generating the increments of α-stable subordinator $D_{\alpha_{1}}\left(t\right)$.

**Step-3**: for generating the increments of TSS $D_{\alpha_{1},\mu_{1}}\left(t\right)$ with pdf $f_{\alpha_{1},\mu_{1}}\left(x,t\right)$, we use the following steps, called “acceptance-rejection method”;
  (a) generate the stable random variable $D_{\alpha_{1}}\left(dt\right)$;
  (b) generate uniform (0,1) rv *W* (independent from $D_{\alpha_{1}}$);
  (c) if $W\leq e^{-\mu_{1}D_{\alpha_{1}}\left(dt\right)}$, then $D_{\alpha_{1},\mu_{1}}\left(dt\right)=D_{\alpha_{1}}\left(dt\right)$ (“accept"); otherwise, go back to (a) (“reject"). Note that, here we used that $f_{\alpha_{1},\mu_{1}}\left(x,t\right)=e^{-\mu_{1}x+\mu_{1}^{\alpha_{1}}t}f_{\alpha_{1}}\left(x,t\right),$ which implies $\frac{f_{\alpha_{1},\mu_{1}}\left(x,t\right)\left(x,dt\right)}{cf_{\alpha_{1}}\left(x,dt\right)}=e^{-\mu_{1}x}$ for $c=e^{{\mu_{1}}^{\alpha_{1}}dt}$ and the ratio is bounded between 0 and 1;
**Step-4**: generate Poisson distributed rv $N(D_{\alpha_{1},\mu_{1}}\left(dt\right))$ with parameter $\lambda_{1}D_{\alpha_{1},\mu_{1}}\left(dt\right)$
**Step-5**: cumulative sum of increments gives the tempered space fractional Poisson process $N_{1}\left(D_{\alpha_{1},\mu_{1}}\left(t\right)\right)$ sample trajectories; and,
**Step-6**: similarly generate $N_{2}\left(D_{\alpha_{2},\mu_{2}}\left(t\right)\right)$, then take difference of these to get the sample paths of the TSFSP$S_{\alpha_{1},\alpha_{2}}^{\mu_{1},\mu_{2}}\left(t\right)$.

The tail probability of α-stable subordinator behaves asymptotically as (see e.g., [30])

$$P\left(D_{\alpha }\left(t\right)>x\right)∼\frac{t}{\Gamma (1-\alpha )}x^{-\alpha },\;\mathrm{as}\;x\to \infty .$$

**Figure 1:**
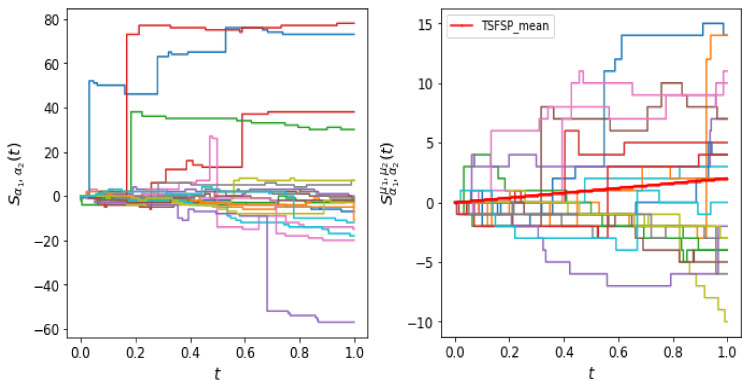
The left hand figure shows the sample trajectories of SFSP with parameters $\alpha_{1}=0.6$, $\alpha_{2}=0.9$, $\lambda_{1}=6$ and $\lambda_{2}=10$. The sample trajectories of TSFSP are shown in the right figure with parameters $\alpha_{1}=0.6$, $\alpha_{2}=0.9$, $\lambda_{1}=6$, $\lambda_{2}=10$, $\mu_{1}=0.2$ and $\mu_{2}=0.5$.

For $\alpha_{1}=0.6$ and $\alpha_{2}=0.9$ and fixed *t*, it is more probable that the value of the rv $D_{\alpha_{1}}\left(t\right)$ is higher than the rv $D_{\alpha_{2}}\left(t\right)$. Thus, for same intensity parameter λ for Poisson process the process $N(D_{\alpha_{1}}\left(t\right))$ will have generally more arrivals than the process $N(D_{\alpha_{2}}\left(t\right))$ until time *t*. This is evident from the trajectories of the SFSP in Figure 1, because the trajectories are biased towards positive side. The TSFPP is a finite mean process, however SFPP is an infinite mean process and hence SFSP paths are expected to have large jumps, since there could be a large number of arrivals in any interval.
